# *Lycium barbarum* Polysaccharide Promotes Maturation of Dendritic Cell via Notch Signaling and Strengthens Dendritic Cell Mediated T Lymphocyte Cytotoxicity on Colon Cancer Cell CT26-WT

**DOI:** 10.1155/2018/2305683

**Published:** 2018-01-28

**Authors:** Wei Wang, Mingxing Liu, Yang Wang, Tao Yang, Dongsheng Li, Feng Ding, Hongzhi Sun, Guang Bai, Qing Li

**Affiliations:** ^1^Department of General Surgery, The First Affiliated Hospital of Jinzhou Medical University, Jinzhou, Liaoning 121001, China; ^2^Department of Internal Medicine, The Third Affiliated Hospital of Jinzhou Medical University, Jinzhou, Liaoning 121001, China

## Abstract

*Lycium barbarum* polysaccharide (LBP) is the major function component of* Lycium barbarum* L. and has been previously reported to induce the phenotypic and functional maturation of dendritic cells (DCs) as well as activating T lymphocytes. In the current study, the immunologic cytotoxicity promoting effect of LBP was assessed and the underlying mechanism was explored. The impact of LBP on the phenotype, maturation, and immunogenicity of DCs was assessed. The activity of Notch pathway which is involved in the regulation of LBP on DCs was detected. Afterwards, the influence of LBP on cytotoxicity of DC-mediated cytotoxicity T lymphocytes (CTLs) to CT26-WT colon cancer cells was further assessed. Administration of LBP induced the phenotypic and functional maturation of DCs. After being subjected to LBP, the expression of Notch and Jagged and Notch targets Hes1 and Hes5 was all upregulated. The cytotoxicity of DC-mediated CTLs was strengthened by administration of LBP. Additionally, cytotoxicity of DC-mediated CTLs on CT26-WT colon cancer cells also increased with effector-target ratio. In conclusion, LBP could induce the phenotypic and functional maturation of DCs via Notch signaling and promote the cytotoxicity of DC-mediated CTLs, which could be employed as a promising adjuvant for cancer immunotherapy.

## 1. Introduction

The concept of immune contexture is mainly emerged based on studies that are mostly performed with human colorectal cancer (CRC) [1]. Study of Pagès et al. showed that high density of intratumoral memory T cells was closely associated with the disease free time and overall survival rate of CRC patients [2]. In addition to the findings in CRC, Becht and his colleagues also proved the beneficial effect of high densities of T cells with a Th1 orientation and of cytotoxic CD8^+^ T cells in non-small cell lung cancer (NSCLC) [3, 4]. Therefore, harnessing the immune system of cancer patients (immunotherapy in oncology) has been proposed as promising therapeutic approach for treatment of types of cancers.

Generally, the immune system can prevent the development and progression of cancer through a mechanism called immune surveillance. However, once the process of immune surveillance fails due to variable causable factors [5], it will lead to the immune selection of tumor cell variants with reduced immunogenicity and allow progression of tumors [6, 7]. Currently, it is commonly recognized that functioning of an effective immunotherapy majorly depends on the roles of immune cells playing in the development of tumors, especially those with antigen presentation capability, that is, dendritic cells (DCs) [8], and those with cytolytic activity, that is, cytotoxicity T lymphocytes (CTLs) [9]. Thus, efficient induction of antitumor immunity via DC-based methods is emerging in recent decades [10–12]. However, induced maturation of DC by microbial products (such as LPS) or inflammatory cytokines (such as TNF) [13–15] is toxic and application limited. Therefore, exploration of nontoxic vehicles that are capable of inducing DC maturation and immunogenicity is imperative.

*Lycium barbarum* L. is a well-recognized East Asia herbal medicine used for treatment and prevention of disease such as insomnia, liver dysfunction, diabetes, visual degeneration, and cancer [16]. The bioactivity of* Lycium barbarum* L. is mainly attributed to its polysaccharide-protein complex [17–19]. The major component of* Lycium barbarum* polysaccharide (LBP) consists of six monosaccharides (galactose, glucose, rhamnose, arabinose, mannose, and xylose) and 18 amino acids [16]. Recent studies infer that LBP can also enhance the immune function of patients [18, 19]. Verified by several studies, administration of LBP can induce the phenotypic and functional maturation and immunogenicity of DCs as well as activating T lymphocytes [16, 20, 21]. Nevertheless, even with the confirmation of the potency of LBP in promoting efficiency of immunotherapy, the underlying mechanism through which LBP modulates the function of DCs and CTLs remains unrevealed.

Thus, in the current study, the impact of LBP on the phenotype, maturation, and immunogenicity of DCs isolated from mice was investigated. To reveal the pathways involved in the regulating effect of LBP on DCs, the expression of Notch pathway members was quantified. The Notch pathway is reported to be a key signaling transduction in the differentiation of DCs [22, 23]. Thereafter, the influence of LBP on cytotoxicity of DC-mediated CTLs to CT26-WT colon cells was further assessed. Findings outlined in the current study showed that administration of LBP promoted the maturation and immunogenicity of DCs via Notch signaling, which strengthened the cytotoxicity of DC-mediated CTLs to tumor cells.

## 2. Materials and Methods

### 2.1. Chemicals, Cell Culture, and Animals

LBPs (purity: 84.32%) for clinical application with an endotoxin content < 0.1 Eu/mg were purchased from Pharmagenesis Inc. (Newtown, PA, USA). Antibody against Notch was purchased from Abcam (Catl. number ab52627, Cambridge, MA). Antibody against Jagged was purchased from Bioss (Catl. number bs-1448R, Beijing, China). Murine colon cancer cell line CT26-WT was obtained from Chi Scientific (Shanghai, China) and cultured in DMEM supplemented with 10% FBS and penicillin/streptomycin at 37°C in an atmosphere of 5%  CO_2_ and 95% air until a density that allowed cell division throughout the course of the experiment. BALB/c mice were purchased from Changsheng Biotechnology (Liaoning, China) and maintained in cages at room temperature (20–25°C) with a constant humidity (55 ± 5%) with access to food and water ad libitum in a 12 : 12-h light-dark cycle. All animal experiments were conducted in accordance with the Guide for the Care and Use of Laboratory Animals published by the National Research Council and under IACUC approval. The use of animals was reviewed and approved by the Animal Care Review Committee of The First Affiliated Hospital of Jinzhou Medical University.

### 2.2. Collection of Bone Marrow Cells (BMCs) and Induction of DCs

The mice were anesthetized with overdose pentobarbital sodium and sacrificed by neck removing method and sterilized with 75% alcohol for 10 min. Thereafter, humerus and tibia were removed and administrated with 75% alcohol for 2 min. Then the marrow cavities of tissues were washed with 1640 medium and BMCs in the suspensions were separated with 70 *μ*m Cell Strainers and centrifuged with Tris-NH_4_Cl Lysis Buffer to collect BMCs for induction of DCs. The collected BMCs incubated with 1640 medium supplemented with 10% fetal bovine serum (FBS) and with 20 ng/ml mGM-CSF and 20 ng/ml rmIL-4 at 37°C in an atmosphere of 5%  CO_2_ and 95% air for 3 days. Afterwards, the suspension was replaced with fresh medium and suspended cells were discarded. After another 5 days, the DCs were collected, centrifuged, and resuspended in 1640 medium for subsequent assays.

### 2.3. LBP Administration

For assessing the effect of LBP on the phenotype, maturation, and immunogenicity of DCs, 5 ml healthy DCs (5 × 10^5^/ml) were cultured in 60 mm plates administrated with different doses of LBP (0 *μ*g/ml, 1 *μ*g/ml, 10 *μ*g/ml, and 100 *μ*g/ml) for different time courses (24 h and 48 h). The treatment combination with the strongest DC maturation inducing effect was employed for subsequent assays.

### 2.4. Isolation of T Lymphocytes from Mice and Induction of CTLs by DCs

T lymphocytes were isolated from spleen of mice: spleen tissues were grinded and cells were suspended by PBS. The mixture was filed with 74-*μ*m filter gauze and then subjected to centrifugation for at 1000 rpm for 10 min to discard supernate. Then the precipitation was added with 2 ml RBC Lysis Buffer and incubated at room temperature for 5 min. After being subjected to centrifugation at 1000 rpm for 10 min, the precipitation was resuspended using 1640 medium. Afterwards, the suspension was mixed with equal volume of lymphocyte separation medium and then centrifuged at 2500 rpm for 25 min. The interlayer of the suspension was collected, resuspended using PBS, and centrifuged at 1000 rpm for 5 min. The precipitation was resuspended using 1640 medium and incubated with DCs or LBP treated DCs at a ratio of 5 : 1 (2 × 10^6^ DCs and 1 × 10^7^ T lymphocytes) in solution containing 500 IU/ml IL-2 and 50 ng/ml CD3 for 4 days [9]. Then the CD8+ CTLs were purified by positive selection using Cell Isolation Kit (Catl. number 130-049-401, Miltenyi Biotec, China) according to the manufacturers' instruction. The phenotypic characteristics of CD8+ CTLs were assessed by detecting expression status of CD3 and CD8 by flow cytometry as described below.

### 2.5. Cytotoxicity Assessment of CTLs on CT26-WT Cells

CT26-WT cells were employed as target cells for assessment of cytotoxicity of different CTLs (effector cells). For ELISA detection of IFN-*γ*, two groups were classified: (A) T + E group, CT26-WT cells treated with CTLs (effector-target ratio: 100 : 1) for 20 h, and (B) T + E + LBP group, CT26-WT cells treated with CTLs (effector-target ratio: 100 : 1) and 100 ng/ml LBP for 20 h. For flow cytometry detection of cell apoptosis, six groups (two groups for effector-target ratio) were set up: (A) T + E group, CT26-WT cells treated with CTLs (effector-target ratio: 10 : 1, 20 : 1, 40 : 1, respectively) for 20 h, and (B) T + E + LBP group, CT26-WT cells treated with CTLs (effector-target ratio: 10 : 1, 20 : 1, and 40 : 1, resp.) and 100 *μ*g/ml LBP for 20 h. For NK activity determination by MTT assay, 18 groups (six groups for effector-target ratio) were classified: (A) T group, health CT26-WT cells, (B) E group, CTLs (effector-target ratio: 10 : 1, 20 : 1, and 40 : 1, resp.), (C) T + E group, CT26-WT cells treated with CTLs (effector-target ratio: 10 : 1, 20 : 1, and 40 : 1, resp.) for 20 h, (D) T + LBP group, health CT26-WT cells treated with 100 *μ*g/ml LBP for 20 h, (E) E + LBP group, CTLs (effector-target ratio: 10 : 1, 20 : 1, and 40 : 1, resp.) treated with 100 ng/ml LBP for 20 h, and (F) T + E + LBP group, CT26-WT cells treated with CTLs (effector-target ratio: 10 : 1, 20 : 1, and 40 : 1, resp.) and 100 *μ*g/ml LBP for 20 h.

### 2.6. Phenotypic and Maturation Assessment of DCs

Upon completion of the 48 h culture, cells in different groups were collected. The morphology of DCs was observed under inverted phase contrast microscope (AE31, Motic, Xiamen, China) at 400x magnification. Then the expressions of CD80 and CD86 on DCs were detected with flow cytometry.

### 2.7. Enzyme-Linked Immunosorbent Assay (ELISA)

Production of TGF-*β*, IL-10, and IL-12 in supernatant of DC cultures (Catl. number EK0515, Catl. number EK0417, and Catl. number EK0422, Boster, China) and production of IFN-*γ* (Catl. number 69-90110, MSK, Wuhan, China) in supernatant were measured using ELISA kits according to the manufacturers' instructions.

### 2.8. Reverse Transcription Real Time PCR (RT^2^-PCR)

Total RNA in cells from different groups was extracted using RNA Purified Total RNA Extraction Kit according to the manufacturer's instruction (Calt. number RP1201, BioTeke, Beijing, China).*β-Actin *was selected as the internal reference gene. RNA was reversely transcribed to cDNA templates using super M-MLV reverse transcriptase (Calt. number RP6502, BioTeke, Beijing, China) and final qPCR reaction mixture (20 *μ*L) consisted of 10 *μ*L of SYBR GREEN mastermix (Catl. number SY1020, Solarbio, China), 0.5 *μ*L of each of the primers (Notch, forward: 5′-CAAGGCACGGAGGAAGAAG-3′ and reverse: 5′-CCAGGTGAGTGTCAGGCATA-3′; Jagged, forward: 5′-CAACGACCGTAATCGCATC-3′ and reverse: 5′-GAAGTGGGCAATCCCTGTG-3′; Hes1, forward: 5′-TGACTGTGAAGCACCTCCG-3′ and reverse: 5′-AAGCGGGTCACCTCGTTCA-3′; Hes5, forward: 5′-CGCTCGCTAATCGCCTCCA-3′ and reverse: 5′-CGGTCCCGACGCATCTTCT-3′;*β-actin* forward: 5′-CTGTGCCCATCTACGAGGGCTAT-3′ and reverse: 5′-TTTGATGTCACGCACGATTTCC-3′), 1 *μ*L of the cDNA template, and 8 *μ*L of Rnase-free H_2_O. Amplification parameters were as follows: denaturation at 95°C for 10 min, followed by 40 cycles at 95°C for 10 s, 60°C for 20 s, and 72°C for 30 s, and the reaction was stopped at 4°C for 5 min. Relative expression levels of the targeted molecules were calculated with Exicycler™ 96 (BIONEER) according to the expression of 2^−ΔΔct^.

### 2.9. Western Blotting Assay

Total protein product was extracted by incubating cells with 1% PMSF-RIPA (Catl. number ST506, Catl. number P0013B, Beyotime Biotechnology, China) and then centrifuged at 12000 rpm for 10 min. *β*-Actin was selected as the internal reference protein. Protein concentrations of different samples were determined using the BCA method (Catl. number P0012, Beyotime Biotechnology, China). 40 *μ*g protein (20 *μ*L volume) was subject to a 10% sodium dodecylsulfate polyacrylamide gel electrophoresis (SDS-PAGE) and transferred onto polyvinylidene difluoride (PVDF) sheets. Then the membranes were washed with TTBS and incubated in skim milk powder solution for 1 h. Primary antibodies against Notch (1 : 1000), Jagged (1 : 500), and *β*-actin (1 : 1000) were incubated with membranes at 4°C overnight. Secondary HRP goat anti-rabbit/goat anti-murine IgG antibodies (1 : 5000) were added to the mixture and incubated with the membranes for 45 min at 37°C. The blots were developed using Beyo ECL Plus reagent. The results were detected in the Gel Imaging System and the relative expression levels of targeted proteins were calculated with Gel-Pro-Analyzer (Media Cybernetics, USA).

### 2.10. MTT Assay

MTT assay was performed to determine the viability of CT26-WT cells in different groups. Briefly, 50 *μ*L exponentially growing cells (2 × 10^5^ cells/ml) were seeded into a 96-well plate in triplicate. Then 5 mg/ml MTT was added to each well and incubated for 4 h at 37°C. The OD values at 490 nm in different wells were recorded using a Microplate Reader. The NK activity was calculated based on the expression: 1 − ((OD_T+E_ − OD_E_)/OD_T_).

### 2.11. Flow Cytometry

Cells in different groups were collected with centrifugation at 1500 rpm for 5 min. The apoptotic rates were measured using an Apoptosis Detection Kit (Catl. number KGA106, KeyGEN BioTECH, Nanjing, China) according to the instructions for manufacturers: briefly, 5 *μ*L Annexin V was added to different wells. After incubation with Annexin V for 10 min at room temperature, the cells were resuspended with 1x binding buffer and added with 5 *μ*L Propidium Iodide (PI). After a 15-min incubation in the dark, the apoptotic rates were analyzed using a FACScan flow cytometry (Accuri C6, BD, USA).

### 2.12. Statistical Analysis

All the data were expressed in the form of mean ± SD. Student's *t*-test, ANOVA, and post hoc tests with Duncan method were performed with a significance level of 0.05 using GraphPad Prism 6 (GraphPad Software, San Diego, CA).

## 3. Results

### 3.1. Morphology and Surface Antibody Characteristics of DCs

**Figure 1:**
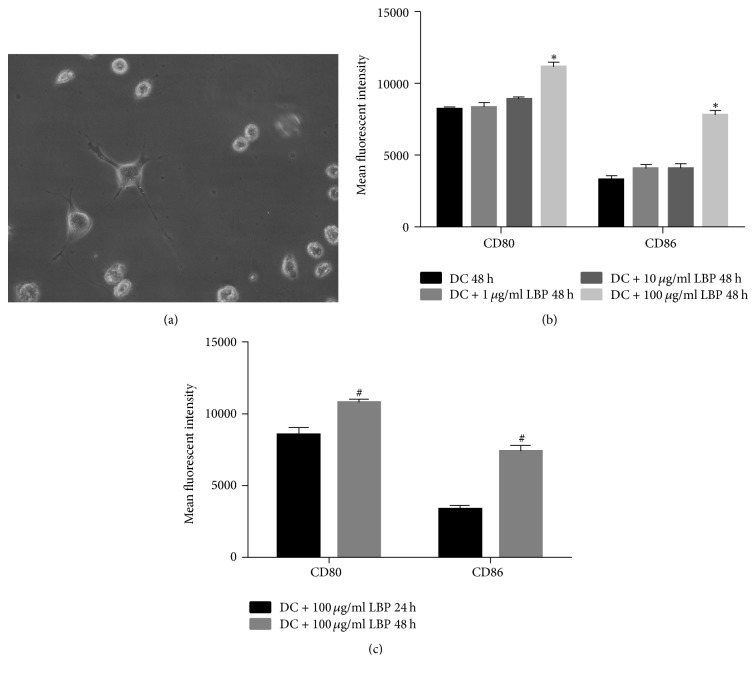
*Morphology and Maturation Detection of BMCs Derived DCs*. (a) Seven days after induction for BMCs, 5 × 10^5^/ml DCs were incubated with LBP at 100 *μ*g/ml for 48 h and cells morphology was detected using an inverted phase contrast microscope. The cells were characterized by long dendrites which are typical features of DCs. (b) 5 × 10^5^/ml DCs were incubated with LBP at different doses (0 *μ*g/ml, 1 *μ*g/ml, 10 *μ*g/ml, and 100 *μ*g/ml) for 48 h. The expression of CD80 and CD86 on DCs was detected using flow cytometry method. The mean fluorescent intensity of CD80 and CD86 was strongest under treatment of 100 *μ*g/ml LBP. (c) 5 × 10^5^/ml DCs were incubated with 100 *μ*g/ml LBP for 24 and 48 h, respectively. The expression of CD80 and CD86 on DCs was detected using flow cytometry method. The mean fluorescent intensity of CD80 and CD86 was strongest after 48 h incubation. Each assay was represented by three independent replicates. ^*∗*^*P* < 0.05 versus the other three groups. ^#^*P* < 0.05 versus 24 h group.

Morphology of DCs was shown in Figure 1(a). Three days after culture, DCs were grown from confluence into suspension. Thereafter, cell colonies were formed by cells with processes on the surface. Seven days after culture, cells were characterized by long dendrites which are typical features of DCs. By employing flow cytometry, the antibodies presented on the surface of the cells were also detected. The administration of LBP further increased the expression of the two antibodies on the cell surface, and the effect was both dose- and time-dependent: with 48 h administration of 100 *μ*g LBP had the strongest inducing effect (Figures 1(b) and 1(c); Figure S1, in Supplementary Material available online at https://doi.org/10.1155/2018/2305683). Therefore, the subsequent assays were performed by incubating DCs with 100 *μ*g LBP for 48 h. The results showed that the DCs were successfully induced from BMCs and that LBP could further promote the maturation process.

### 3.2. Administration of LBP Induced the Production of IL-12 and Suppressed the Production of IL-10 as well as TGF-*β*

**Figure 2:**
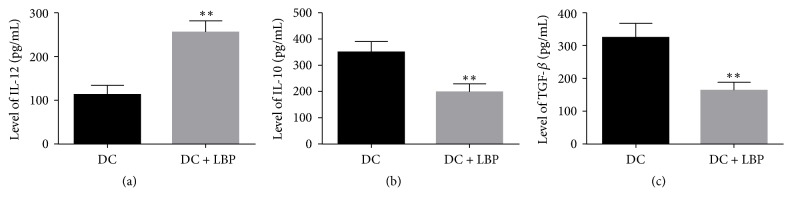
*Quantitative Analysis Results of IL-12, IL-10, and TGF-β Levels*. ((a), (b), (c)) 5 × 10^5^/ml DCs were incubated with LBP at 100 *μ*g/ml for 48 h and production of IL-12, IL-10, and TGF-*β* in supernatant of DC cultures was measured using ELISA kits according to the manufacturers' instructions. Administration of LBP induced the expression of IL-12 while suppressing the expression of IL-10 and TGF-*β*. Each assay was represented by three independent replicates. ^*∗∗*^*P* < 0.01 versus DC group.

The levels of IL-10 and TGF-*β* in the supernatants of DCs were decreased by administration of LBP, and the differences between DC and LBP groups were statistically significant (*P* < 0.05) (Figures 2(a) and 2(b)). In contrast, the levels of IL-12 in the supernatants of DCs were significantly increased (*P* < 0.05) (Figure 2(c)).

### 3.3. Administration of LBP Initiated Notch Signaling in DCs

**Figure 3:**
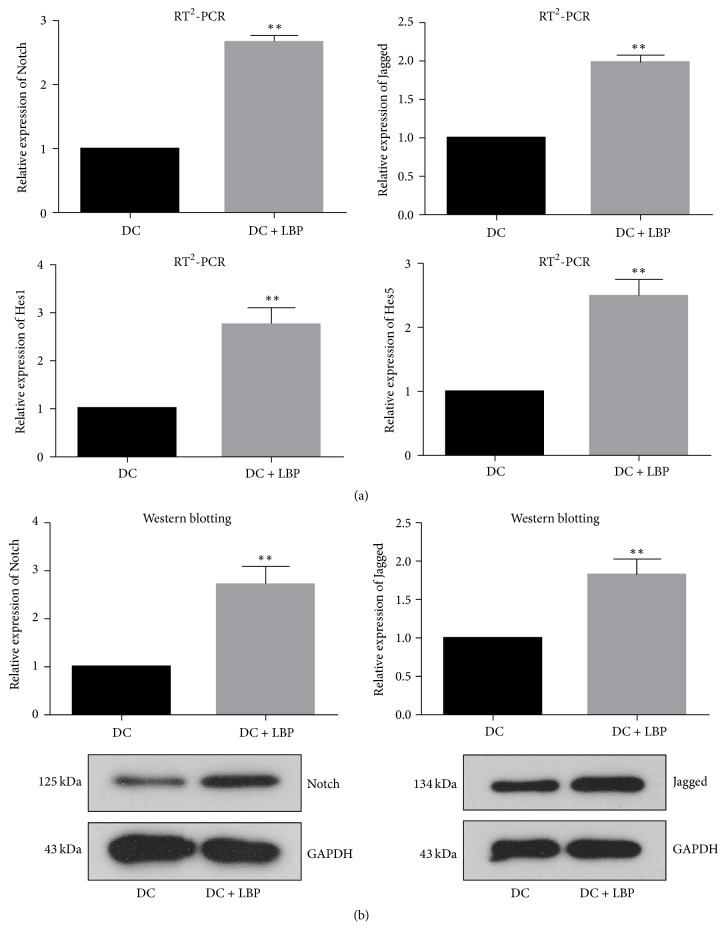
*Detection of Notch Signaling*. (a) 5 × 10^5^/ml DCs were incubated with LBP at 100 *μ*g/ml for 48 h and then subjected to RNA Purified Total RNA Extraction Kit to collect total RNA. The expression levels of Notch, Jagged, Hes1, and Hes5 were detected with RT^2^-PCR method. Administration of LBP up-regulated expression of Notch, Jagged, Hes1, and Hes5 at mRNA level. (b) 5 × 10^5^/ml DCs were incubated with LBP at 100 *μ*g/ml for 48 h. Then cells were incubated with 1% PMSF-RIPA and the centrifuged at 12000 rpm for 10 min to collect total protein. The expression levels of Notch and Jagged were detected with western blotting assay. Administration of LBP up-regulated expression of Notch and Jagged at protein. Each assay was represented by three independent replicates. ^*∗∗*^*P* < 0.01 versus DC group.

To uncover the mechanism which drives the function of LBP on DCs, the expression of Notch and Jagged was detected both at mRNA and at protein levels. The expression of targets of Notch, including Hes1 and Hes5, was detected with RT^2^-PCR. It was found that incubation with LBP enhanced the expression of all indicators (Figures 3(a) and 3(b)). Given the fact that Notch signaling is crucial to the differentiation of DCs, the results of molecular detection confirmed the conclusion that LBP was capable of inducing the differentiation of DCs as illustrated by morphology and antibodies expression detection, which depended on Notch signaling activation.

### 3.4. Administration of LBP Strengthened the Cytotoxicity of DC-Mediated CTLs on Colon Cancer Cell CT26-WT

**Figure 4:**
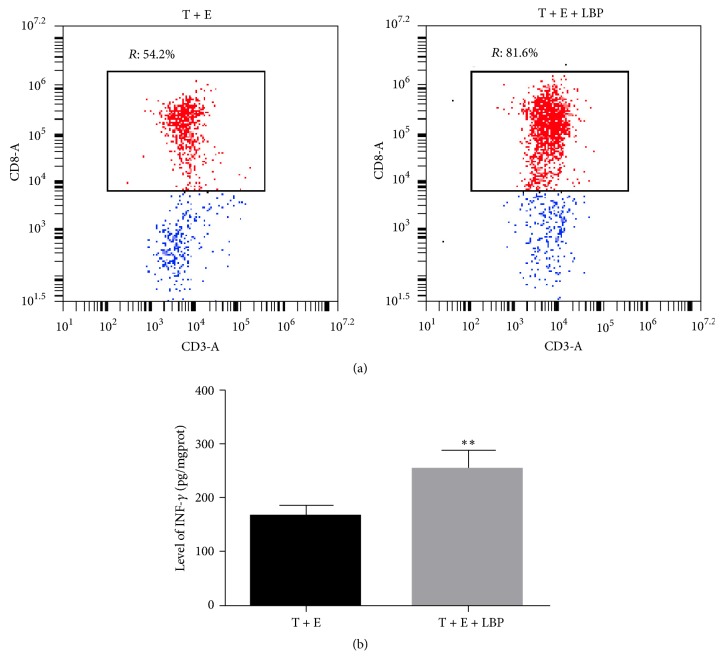
*Detection of DC-Mediated CTLs' Differentiation*. (a) T cells were incubated with DCs or LBP treated DCs at a ratio of 5 : 1 (2 × 10^6^ DCs and 1 × 10^7^ T lymphocytes) for 4 days. Then the CD8+ CTLs were purified by positive selection using Cell Isolation Kit. The phenotypic characteristics of CD8+ CTLs were assessed by detecting expression status of CD3 and CD8 by flow cytometry. The proportion of CD3^+^CD8^+^ T lymphocytes was increased in T + E + LBP group. (b) CT26-WT cells were cells treated with CTLs (effector-target ratio: 100 : 1) for 20 h or CTLs (effector-target ratio: 100 : 1) and 100 ng/ml LBP for 20 h. Production of IFN-*γ* in T cells was detected with ELISA assay. Production of IFN-*γ* was increased in T + E + LBP group. Each assay was represented by three independent replicates. ^*∗∗*^*P* < 0.01 versus T + E group.

**Figure 5:**
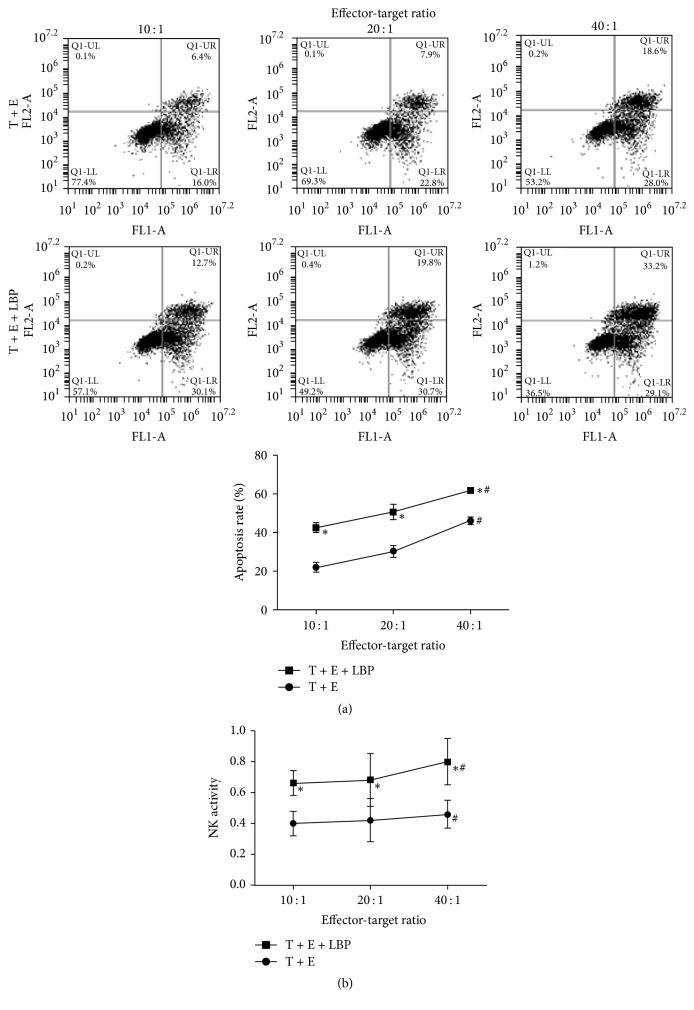
*Detection of Cytotoxicity of CTLs*. (a) CT26-WT cells treated with CTLs (effector-target ratio: 10 : 1, 20 : 1, and 40 : 1, resp.) for 20 h or with CTLs (effector-target ratio: 10 : 1, 20 : 1, and 40 : 1, resp.) and 100 *μ*g/ml LBP for 20 h. The apoptosis rate was detected with flow cytometry. The cell apoptosis rate was increased by LBP administration and effector-target ratio. (b) CT26-WT cells, CTLs, and LBP were incubated in different combinations for 20 h [CT26-WT cells, CTLs, and CT26-WT cells + CTLs (effector-target ratio: 10 : 1, 20 : 1, and 40 : 1), CT26-WT cells + 100 *μ*g/ml LBP, CTLs + 100 *μ*g/ml LBP, and CT26-WT cells + CTLs (effector-target ratio: 10 : 1, 20 : 1, and 40 : 1) + 100 *μ*g/ml LBP]. The viability of CT26-WT cells was detected with MTT assay and represented by OD values at 490 nm. The NK activity was calculated based on the expression: 1 − ((OD_T+E_ − OD_E_)/OD_T_). NK activity of CTLs was increased by LBP administration and effector-target ratio. Each assay was represented by six independent replicates ^*∗*^*P* < 0.05 versus T + E group, *P* < 0.05. ^#^*P* < 0.05 versus 10 : 1 and 20 : 1 effector-target ratio.

CTLs were successfully induced by DC incubation and the cytotoxicity characteristics of DC-mediated CTLs were even strengthened by administration of LBP: the proportion of CD3^+^CD8^+^ cells significantly increased after being subjected to LBP for 4 days (54.5 ± 4.26 versus 80.9 ± 7.93) (Figure 4(a)). Moreover, the production of IFN-*γ* by CTLs was also augmented by administration of LBP, representing the promising potential of LBP to promote immunologic cytotoxicity (Figure 4(b)). As a result, the cytotoxicity of CTLs on CT26-WT cells was increased: the total apoptotic rates of CT26-WT cell in LBP treated group and the NK activity of LBP treated CTLs were significantly increased compared with normal CTLs (*P* < 0.05) (Figure 5). Additionally, effector-target ratio also served as a factor that influenced the cytotoxicity of CTLs: although no significant difference was detected between effector-target ratios 10 : 1 and 20 : 1, the apoptotic rate and NK activity in 40 : 1 group were dramatically higher than the other two groups (*P* < 0.05).

## 4. Discussion

The application of natural compounds as potential improvements for human health keeps increasing in popularity in recent years. Since ancient years, oriental herbal medicine has been used for treatment of malignancy in Eastern countries. LBP, which is the biologically active compounds of* Lycium barbarum* L., is proved to have biological activities such as anticancer, antioxidant, hypoglycemic, and immunological activities [24–26]. Previous studies have shown that administration of LBP induces cell cycle arrest in colon cancer [27] and promotes the maturation of DCs as well as increasing cytotoxicity of CTLs [16, 20, 21]. The findings of the previous studies took a step further in the current study: after confirming the inducing effect of LBP on DCs, the mechanism involved in the function of LBP on DCs and its effect on DC-mediated CTL cytotoxicity on tumor cells were also explored. Our results showed that LBP treatment activated the Notch signaling in DCs, representing the pathway through which LBP promoted DCs maturation. Moreover, the effect of LBP on the production of IL-10, IL-12, and TGF-*β* might explain the mechanism driving the LBP function on activation and function differentiation of CTLs.

It was demonstrated in the current study that LBP treatment upregulated the expression of CD80 and CD86 on BMC derived DCs, suggesting that LBP induced the phenotypic maturation of DCs. Moreover, LBP also promoted function maturation of DCs, which was validated by the augmented production of IL-12 and suppressed production of IL-10 and TGF-*β*. IL-12 is a functional DC maturation marker with a molecular mass of 70 kDa, which is a potent IFN-*γ* inducer for CTLs [28, 29]. To the contrary, IL-10 and TGF-*β* mediate active suppression of specific T lymphocytes responses as an essential mechanism in immune response [30]. The increased IL-12 and decreased IL-10 and TGF-*β*, along with the enhanced DC activation by LBP treatment, would promote the IFN-*γ*-secreting T cell differentiation following T cell activation [9]. Indeed, in ELSIA assay, the production of IFN-*γ* was significantly upregulated. Previous studies have demonstrated that IFN-*γ*^+^ CTLs are critical for antitumor immunotherapies [9]. Therefore, the enhanced DC activation and higher frequency of IFN-*γ*^+^ CTLs evidently indicated that LBP was a promising adjuvant therapeutic modality along with immunotherapies.

The pathway through which LBP exerted its function on DC maturation was also preliminarily investigated in the current study. It was found that administration of LBP would induce the activation of Notch signaling. The differentiation of DC is modulated via a network of soluble and cell-bound factors in stroma [22]. Notch signaling is one of the most major components of the process. Generally, Notch signaling is initiated upon the binding of the extracellular domain of Notch receptors to Notch ligands: Jagged and Delta. With regard to DC differentiation, function of Notch signaling is exerted through its cooperation with Wnt pathways [22]. Then the activated Wnt pathway will result in sustained upregulation of IL-12 [31]. However, the inhibition of IL-10 and TGF-*β* associated with the activated Notch signaling is confusing in that the activation of Notch signaling in DCs always induces the production of IL-10 by T_H_1 cells. And activated Wnt signaling due to Notch activation is also reported to upregulate TGF-*β* signaling during myofibroblast differentiation [32–34]. Subjecting DCs to LBP in the current study seemed to lead to a contrary conclusion to previous studies, which made it hard to evidently elaborate the mechanism through which LBP affected DC maturation and cytokines secretion. Given that the activated Notch signaling was associated with decreased IL-10 and TGF-*β*, it was likely that the modulating effect of LBP on DCs comprised more complicated pathways as we had expected in the first place.

## 5. Conclusions

Being widely used as an injection in clinical patients in China to improve immune functions, LBP has showed its promising potential for further exploration as an effective adjuvant for cancer immunotherapy. In the current study, administration of LBP not only induced the phenotypic and functional maturation of DCs but also promoted the cytotoxicity of DC-mediated CTLs. We also attempted to preliminarily study the pathways involved in the effect of LBP on DC, but the activated Notch signaling due to LBP treatment introduced more questions to the mechanism related to LBP function on DCs. To comprehensively understand the effect of LBP on immune responses, more work will be needed in the future.

## Supplementary Material

Figure S1. Maturation detection of BMCs derived DCs. A, 5 × 105/ml DCs were incubated with LBP at different doses (0 μg/ml, 1 μg/ml, 10 μg/ml, and 100 μg/ml) for 48 h. The expression of CD80 and CD86 on DCs was detected using flow cytometry method. The numbers of CD80 and CD86 positive cells were highest under treatment of 100 μg/ml LBP. B, 5 × 105/ml DCs were incubated with 100 μg/ml LBP for 24 and 48 h, respectively. The expression of CD80 and CD86 on DCs was detected using flow cytometry method. The numbers of CD80 and CD86 positive cells were highest after 48-hour incubation. Each assay was represented by three independent replicates.

## References

[1] Becht E., Giraldo N. A., Dieu-Nosjean M.-C., Sautès-Fridman C., Fridman W. H. (2016). Cancer immune contexture and immunotherapy. *Current Opinion in Immunology*.

[2] Coussens L. M., Werb Z. (2002). Inflammation and cancer. *Nature*.

[3] Dieu-Nosjean M.-C., Antoine M., Danel C. (2008). Long-term survival for patients with non-small-cell lung cancer with intratumoral lymphoid structures. *Journal of Clinical Oncology*.

[4] Goc J., Germain C., Vo-Bourgais T. K. D. (2014). Dendritic cells in tumor-associated tertiary lymphoid structures signal a Th1 cytotoxic immune contexture and license the positive prognostic value of infiltrating CD8^+^ T cells. *Cancer Research*.

[5] Swann J. B., Smyth M. J. (2007). Immune surveillance of tumors. *The Journal of Clinical Investigation*.

[6] Dunn G. P., Old L. J., Schreiber R. D. (2004). The immunobiology of cancer immunosurveillance and immunoediting. *Immunity and Immunoediting*.

[7] Pernot S., Terme M., Voron T. (2014). Colorectal cancer and immunity: what we know and perspectives. *World Journal of Gastroenterology*.

[8] Kumar J., Kale V., Limaye L. (2015). Umbilical cord blood-derived CD11c^+^ dendritic cells could serve as an alternative allogeneic source of dendritic cells for cancer immunotherapy. *Stem Cell Research & Therapy*.

[9] Liu Y., Tian X., Jiang S. (2015). Umbilical cord blood-derived dendritic cells infected by adenovirus for SP17 expression induce antigen-specific cytotoxic T cells against NSCLC cells. *Cellular Immunology*.

[10] Fong L., Engleman E. G. (2000). Dendritic cells in cancer immunotherapy. *Annual Review of Immunology*.

[11] Brossart P., Wirths S., Brugger W., Kanz L. (2001). Dendritic cells in cancer vaccines. *Experimental Hematology*.

[12] Banchereau J., Schuler-Thurner B., Palucka A. K., Schuler G. (2001). Dendritic cells as vectors for therapy. *Cell*.

[13] Sallusto F., Lanzavecchia A. (1994). Efficient presentation of soluble antigen by cultured human dendritic cells is maintained by granulocyte/macrophage colony-stimulating factor plus interleukin 4 and downregulated by tumor necrosis factor alpha. *The Journal of Experimental Medicine*.

[14] Winzler C., Rovere P., Rescigno M. (1997). Maturation stages of mouse dendritic cells in growth factor-dependent long-term cultures. *The Journal of Experimental Medicine*.

[15] Roake J. A., Rao A. S., Morris P. J., Larsen C. P., Hankins D. F., Austyn J. M. (1995). Dendritic cell loss from nonlymphoid tissues after systemic administration of lipopolysaccharide, tumor necrosis factor, and interleukin 1. *The Journal of Experimental Medicine*.

[16] Chen Z., Lu J., Srinivasan N., Tan B. K. H., Chan S. H. (2009). Polysaccharide-protein complex from Lycium barbarum L. is a novel stimulus of dendritic cell immunogenicity. *The Journal of Immunology*.

[17] Huang L., Lin Y., Tian G., Ji G. (1998). [Isolation, purification and physico-chemical properties of immunoactive constituents from the fruit of Lycium barbarum L.]. *Yao Xue Xue Bao = Acta Pharmaceutica Sinica*.

[18] Gan L., Zhang S.-H., Liu Q., Xu H.-B. (2003). A polysaccharide-protein complex from Lycium barbarum upregulates cytokine expression in human peripheral blood mononuclear cells. *European Journal of Pharmacology*.

[19] Gan L., Zhang S. H., Yang X. L., Xu H. B. (2004). Immunomodulation and antitumor activity by a polysaccharide-protein complex from *Lycium barbarum*. *International Immunopharmacology*.

[20] Chen Z., Kwong Huat Tan B., Chan S. H. (2008). Activation of T lymphocytes by polysaccharide-protein complex from Lycium barbarum L.. *International Immunopharmacology*.

[21] Zhu J., Zhao L.-H., Zhao X.-P., Chen Z. (2007). Lycium barbarum polysaccharides regulate phenotypic and functional maturation of murine dendritic cells. *Cell Biology International*.

[22] Zhou J., Cheng P., Youn J.-I., Cotter M. J., Gabrilovich D. I. (2009). Notch and wingless signaling cooperate in regulation of dendritic cell differentiation. *Immunity*.

[23] Cheng P., Nefedova Y., Corzo C. A., Gabrilovich D. I. (2007). Regulation of dendritic-cell differentiation by bone marrow stroma via different Notch ligands. *Blood*.

[24] Li X.-M. (2007). Protective effect of Lycium barbarum polysaccharides on streptozotocin-induced oxidative stress in rats. *International Journal of Biological Macromolecules*.

[25] Li X. M., Ma Y. L., Liu X. J. (2007). Effect of the *Lycium barbarum* polysaccharides on age-related oxidative stress in aged mice. *Journal of Ethnopharmacology*.

[26] Wu H., Guo H., Zhao R. (2006). Effect of Lycium barbarum polysaccharide on the improvement of antioxidant ability and DNA damage in NIDDM rats. *Yakugaku Zasshi*.

[27] Mao F., Xiao B., Jiang Z., Zhao J., Huang X., Guo J. (2011). Anticancer effect of Lycium barbarum polysaccharides on colon cancer cells involves G0/G1 phase arrest. *Medical Oncology*.

[28] Jung D., Jeong S. K., Lee C.-M. (2011). Enhanced efficacy of therapeutic cancer vaccines produced by Co-treatment with mycobacterium tuberculosis heparin-binding hemagglutinin, a novel TLR4 agonist. *Cancer Research*.

[29] Noh K. T., Shin S. J., Son K. H. (2012). The mycobacterium avium subsp. paratuberculosis fibronectin attachment protein, a toll-like receptor 4 agonist, enhances dendritic cell-based cancer vaccine potency. *Experimental & Molecular Medicine*.

[30] Jutel M., Akdis M., Budak F. (2003). IL-10 and TGF-*β* cooperate in the regulatory T cell response to mucosal allergens in normal immunity and specific immunotherapy. *European Journal of Immunology*.

[31] George S. J. (2008). Wnt pathway: A new role in regulation of inflammation. *Arteriosclerosis, Thrombosis, and Vascular Biology*.

[32] Rutz S., Janke M., Kassner N., Hohnstein T., Krueger M., Scheffold A. (2008). Notch regulates IL-10 production by T helper 1 cells. *Proceedings of the National Acadamy of Sciences of the United States of America*.

[33] Saraiva M., O'Garra A. (2010). The regulation of IL-10 production by immune cells. *Nature Reviews Immunology*.

[34] Carthy J. M., Garmaroudi F. S., Luo Z., McManus B. M. (2011). Wnt3a induces myofibroblast differentiation by upregulating TGF-*β* signaling through SMAD2 in a *β*-catenin-dependent manner. *PLoS ONE*.

