# Supplementary material for: Lycium barbarum Polysaccharide Promotes Maturation of Dendritic Cell via Notch Signaling and Strengthens Dendritic Cell Mediated T Lymphocyte Cytotoxicity on Colon Cancer Cell CT26-WT
Source: Evid Based Complement Alternat Med. 2018 Jan 28;2018:2305683. doi: 10.1155/2018/2305683 (PMC5829330; doi:10.1155/2018/2305683)

**Figure S1 Maturation detection of BMCs derived DCs.** A, 5×10^5^/ml DCs were incubated with LBP at different doses (0 μg/ml, 1 μg/ml, 10 μg/ml, and 100 μg/ml) for 48 h. The expression of CD80 and CD86 on DCs was detected using flow cytometry method. The numbers of CD80 and CD86 positive cells were highest under treatment of 100 μg/ml LBP. B, 5×10^5^/ml DCs were incubated with 100 μg/ml LBP for 24 and 48 h, respectively. The expression of CD80 and CD86 on DCs was detected using flow cytometry method. The numbers of CD80 and CD86 positive cells were highest after 48 incubation. Each assay was represented by three independent replicates.

Figure S1


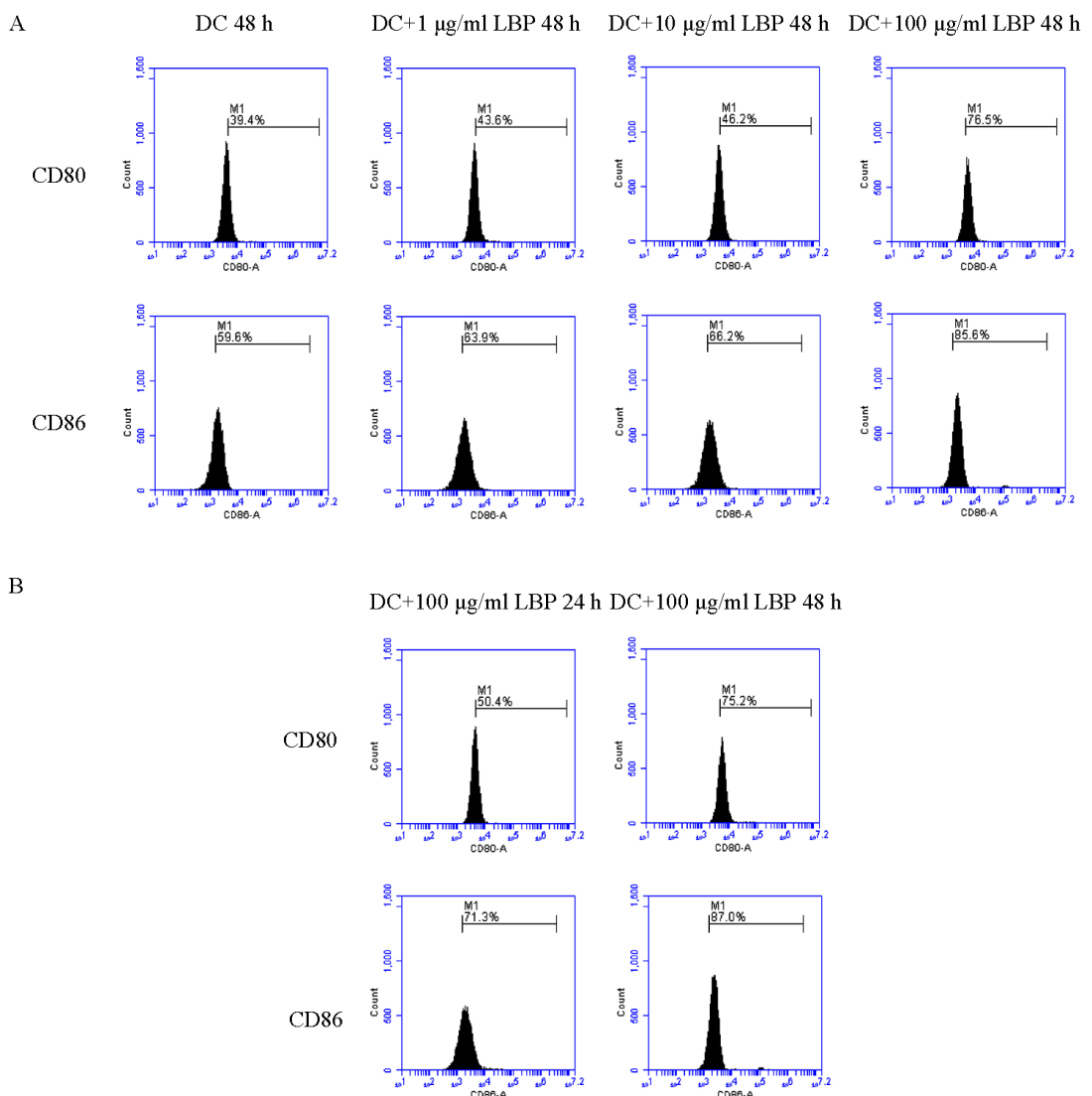

Supplement: Supplementary file 1 — Figure S1. Maturation detection of BMCs derived DCs. A, 5 × 10^5^/ml DCs were incubated with LBP at different doses (0 μg/ml, 1 μg/ml, 10 μg/ml, and 100 μg/ml) for 48 h. The expression of CD80 and CD86 on DCs was detected using flow cytometry method. The numbers of CD80 and CD86 positive cells were highest under treatment of 100 μg/ml LBP. B, 5 × 10^5^/ml DCs were incubated with 100 μg/ml LBP for 24 and 48 h, respectively. The expression of CD80 and CD86 on DCs was detected using flow cytometry method. The numbers of CD80 and CD86 positive cells were highest after 48-hour incubation. Each assay was represented by three independent replicates. [file 2305683.f1.doc]
